# Value of DWI Combined with Magnetic Resonance Spectroscopy in the Differential Diagnosis between Recurrent Glioma and Radiation Injury: A Meta-Analysis

**DOI:** 10.1155/2022/1629570

**Published:** 2022-10-25

**Authors:** Hongyi Li, Yang Duan, Na Liu, Junyi Dong, Yuanzi Liang, Ronghui Ju

**Affiliations:** ^1^Department of Radiology, The People's Hospital of Liaoning Province, Shenyang 110016, China; ^2^Department of Radiology, The People's Hospital of China Medical University, Shenyang 110016, China; ^3^Department of Radiology, The General Hospital of Northern Theater Command, Shenyang 110016, China

## Abstract

To analyse the value of the apparent diffusion coefficient (ADC) in diffusion-weighted imaging (DWI) and the choline (Cho)/creatine (Cr) ratio and Cho/N-acetyl-aspartate (NAA) ratio in magnetic resonance spectroscopy (MRS) in the differential diagnosis between recurrent glioma and radiation injury. Chinese and English studies related to the diagnosis of recurrent glioma and radiation injury using DWI and MRS and published before 15 October 2022 were retrieved from PubMed, Embase, the Cochrane Library, China National Knowledge Infrastructure, China Biomedical Literature Database, VIP Journal Database, and Wanfang Database for a meta-analysis. A total of 11 articles were included in this study. ADC was lower in the recurrent glioma group than in the radiation injury group (standardized mean difference = −1.29, 95% confidence interval (CI) (−1.87, −0.71), *P* < 0.001). The Cho/Cr ratio was higher in the recurrent glioma group than in the radiation injury group (weighted mean difference = 0.65, 95% CI (0.40, 0.90), and *P* < 0.001). The Cho/NAA ratio was higher in the recurrent glioma group than in the radiation injury group, as evidenced by the sensitivity analysis. The sensitivity and specificity of the Cho/Cr ratio were 0.85 (0.73–0.92) and 0.82 (0.67–0.91), respectively, and the area under the curve was 0.86. The sensitivity and specificity of the Cho/NAA ratio were 0.82 (0.66–0.91) and 0.94 (0.69–0.99), respectively, and the area under the curve was 0.93. This meta-analysis showed that ADC, Cho/Cr, and Cho/NAA ratios all had high sensitivity and specificity. Therefore, DWI combined with MRS can effectively improve the diagnosis of recurrent glioma and radiation injury.

## 1. Introduction

Glioma is the most common primary intracranial tumour, and it exhibits infiltrating growth. Therefore, it is often treated with surgical resection, supplemented with postoperative radiotherapy and targeted chemotherapy [1]. Conventional postoperative radiotherapy kills tumour cells and inhibits tumour cell growth while causing brain tissue damage, leading to radiation injury. Clinically, radiation-induced brain injury has become one of the serious complications of radiotherapy. Approximately 20% of patients with glioma will have different degrees of radiation-induced brain injury after radiotherapy. The higher the radiation dose, the earlier the occurrence of radiation-induced brain injury and the more obvious the brain oedema and clinical symptoms [2]. Radiation injury can cause demyelination, degeneration, and even death of glial cells and can damage the blood–brain barrier, so it is difficult to determine whether a new enhanced lesion in the resected site or irradiated site is recurrent glioma or radiation injury by conventional imaging techniques [3, 4].

Diffusion-weighted imaging (DWI) visualizes anatomical structures by detecting the diffuse movement of water molecules and reflects the structural changes of a tissue at the cellular level. A hypointense signal on DWI indicates more diffuse movement of water molecules, apparent diffusion coefficient (ADC), which is the diffusion coefficient of water molecules in a voxela and a commonly used quantitative DWI measure [5]. A higher apparent diffusion coefficient while a hyperintense signal on a DWI image indicates restricted diffusion of water molecules and a lower ADC [6]. Therefore, recurrent glioma and radiation injury can be quantitatively analysed by measuring ADC.

Magnetic resonance spectroscopy (MRS) can quantitatively detect changes in metabolites in tissues in a noninvasive manner [1]. Metabolites commonly detected include N-acetyl-aspartate (NAA), choline (Cho), creatine (Cr), lipids (Lip), and lactic acid (Lac). There are fundamental differences in histology and in cellular metabolites between recurrent glioma and radiation injury. Therefore, MRS can be used to distinguish recurrent glioma and radiation injury through multiparameter comparative analysis [7, 8]. However, it has been found in clinical diagnosis that single-sequence imaging cannot accurately distinguish between glioma recurrence and radiation-induced brain injury due to its limited diagnostic accuracy and the low sensitivity of the measured values. Therefore, it is necessary to combine multisequence and multiparameter imaging to improve the diagnostic accuracy. A previous study analyzeed the specific correlation between diffusion tensor imaging (DTI) -derived metrics and MRS metabolite ratios in the brains of glioblastoma patients and found that peritumoral oedema represents the biggest challenge, with at least ten significant correlations between DTI and MRS that need additional studies [9].

Here, we performed a meta-analysis to systematically evaluate the domestic and international achievements in the differentiation between glioma recurrence and radiation-induced brain injury using MRS combined with DWI. We aimed to provide reliable imaging parameters for the rapid and accurate identification of radiation-induced brain injury and glioma recurrence, thus helping clinicians choose suitable treatment options and improve patient prognosis.

## 2. Materials and Methods

### 2.1. Literature Search

Studies in Chinese and English related to the differential diagnosis between recurrent glioma and radiation injury using DWI combined with MRS and published before 15 October 2022 were retrieved from PubMed, Embase, Cochrane Library, China National Knowledge Infrastructure (CNKI), China Biomedical Literature Database (CBM), VIP Journal Database (VIP), and Wanfang Database. The search terms were “DWI,” “MRS,” “diffusion-weighted imaging,” “magnetic resonance spectroscopy,” “glioma,” and “radiation injury.” The search formula (((glioma) AND (radiation injury)) AND ((magnetic resonance spectroscopy) OR (MRS))) AND ((diffusion-weighted imaging) OR (DWI)) was used to retrieve English studies. The equivalent Chinese terms were used in the Chinese databases.

### 2.2. Inclusion and Exclusion Criteria

Inclusion criteria were as follows: (1) the subjects in the included studies were patients with definite diagnosis of glioma; (2) the diagnostic method in the literature was DWI combined with MRS; (3) the outcome measures of the study included ① ADC, ② Cho/Cr (metabolite Cho/(Cr/phosphocreatine)) ratio, ③ Cho/NAA (metabolite Cho/NAA) ratio, ④ the diagnostic efficacy of the Cho/Cr ratio, and the diagnostic efficacy of the Cho/NAA ratio; (4) the data in the literature were complete.

Exclusion criteria were as follows: (1) incomplete statistical results or incomplete relevant data; (2) repeated publications; (3) diagnostic methods other than DWI combined with MRS; and (4) conference papers, meta-analyses, and literature reviews.

### 2.3. Literature Screening and Data Extraction

Two researchers first independently screened the retrieved studies based on the inclusion and exclusion criteria and then cross-checked the studies. If they had controversial opinions on a paper, the paper was evaluated by a third researcher, and then all three of them would discuss it to reach a consensus. Two researchers extracted relevant data from the included studies, including first author(s), publication year, country of publication, sample size, ADC, the Cho/Cr ratio, the diagnostic efficacy of the Cho/NAA ratio, the Cho/Cr ratio, and the diagnostic efficacy of the Cho/NAA ratio.

### 2.4. Evaluation of Literature Quality

A quality assessment was conducted, adapting to this particular review the Quality Assessment of Diagnostic Accuracy Studies-2 (QUADAS-2) tool. The QUADAS-2 format includes four domains: (1) patient selection; (2) index testing; (3) reference standard; and (4) flow and timing. For each domain, the risk of bias and concerns about applicability (the latter not applying to the domain of flow and timing) were analysed. The results of the quality assessment were used for descriptive purposes to provide an evaluation of the overall quality of the included studies and to investigate potential sources of heterogeneity.

### 2.5. Statistical Methods

All the data were analysed in Stata 16.0. Measurement data are represented as the weighted mean difference (WMD), and the 95% confidence interval (CI) was used as the indicator of the effect size. Interstudy heterogeneity was determined by combing the *χ*^2^ test and *I*^2^ quantitative analysis. If *P* > 0.1 and *I*^2^ < 50%, the interstudy heterogeneity was acceptable, and the fixed-effects model was used for meta-analysis; if *P* < 0.1 and *I*^2^ > 50%, the interstudy heterogeneity was large, and the random-effects model was used for analysis [10].

## 3. Results

### 3.1. Flowchart of Literature Retrieval and Results

**Figure 1:**
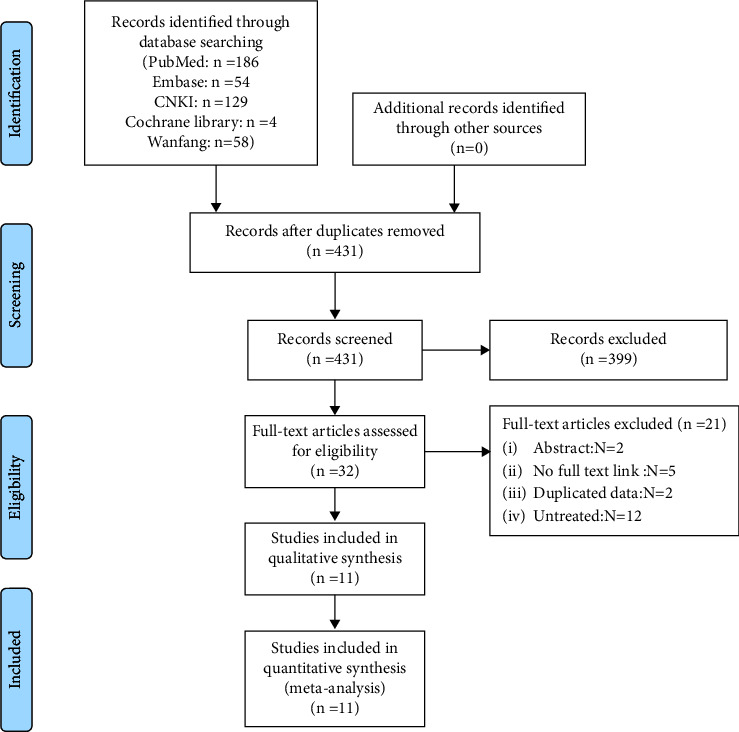
Flowchart of literature screen.

A total of 431 relevant original articles were identified in this meta-analysis. After carefully reading the titles and abstracts and screening the articles according to the inclusion and exclusion criteria, 32 articles were left. Then, after reading the whole articles, 11 available articles were included and all the research was retrospective studies (see Figure 1).

### 3.2. Methodological Quality of Included Studies

**Figure 2:**
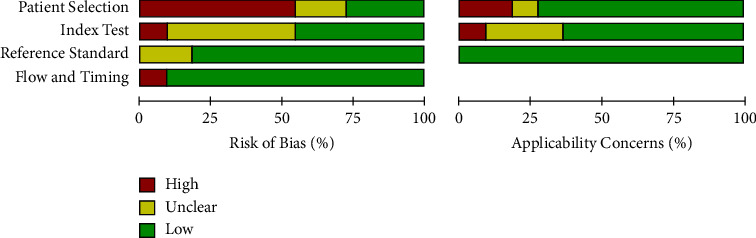
The risk of bias in the studies conducted was measured by using the QUADAS-2 tool.

We extracted this data using a modified QUADAS‐2 criteria proforma that focused on four domains of methodological quality: patient selection; index test; reference standard; and flow and timing. The domain with the highest level of risk for bias across all studies was that of patient selection (>50%) (Figure 2).

### 3.3. Basic Information and Quality Evaluation of the Included Articles

A total of 11 articles were included in this study, involving 320 patients [11–21]. The basic characteristics and quality evaluation of the included studies are given in Table 1.

### 3.4. Meta-Analysis of Relative ADC (rADC)

**Figure 3:**
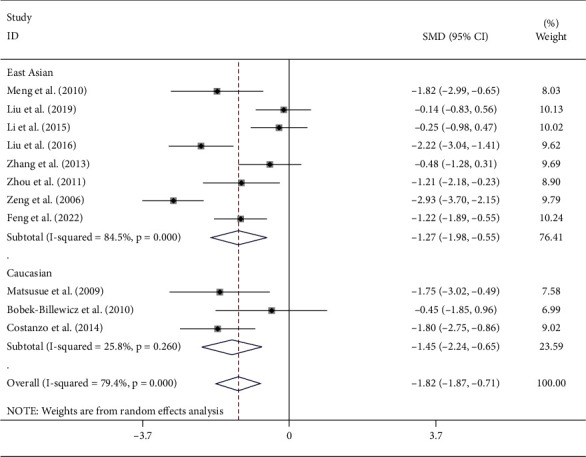
Forest plot of the rADC of the radiation injury group and the recurrent glioma group detected by DWI combined with MRS.

The rADC of the included studies had a high degree of heterogeneity (*I*^2^ = 79.4%, *P* < 0.001). The random-effects model was used to combine the effect sizes. The rADC was significantly lower in the recurrent glioma group than in the radiation injury group (standardized mean difference = −1.29, 95% CI (−1.87, −0.71), *P* < 0.001). Besides, subgroup analysis demonstrated consistent results with the pooled diagnostic performance for both East Asian and Caucasian populations (see Figure 3).

### 3.5. Meta-Analysis of the Cho/Cr Ratio

**Figure 4:**
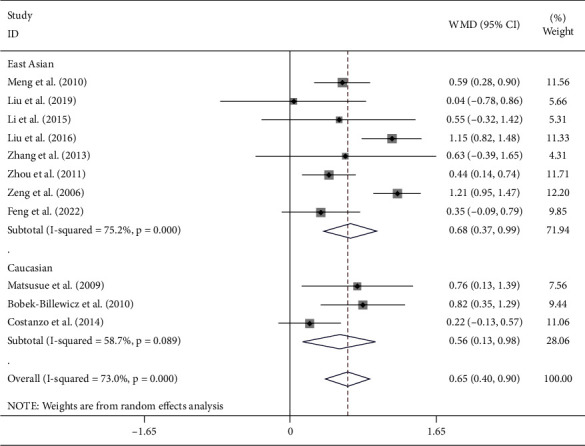
Forest plot of the Cho/Cr ratio of the radiation injury group and the recurrent glioma group detected by DWI combined with MRS.

The Cho/Cr ratio of the included studies had moderate heterogeneity (*I*^2^ = 73.0%, *P* < 0.001). The random-effects model was used to combine the effect size. The Cho/Cr ratio was significantly elevated in the recurrent glioma group than in the radiation injury group (WMD = 0.65, 95% CI (0.40, 0.90), *P* < 0.001). The subgroup analysis based on ethnicity showed that the Cho/Cr ratio was significantly higher in the recurrent glioma group than in those radiation injury group (see Figure 4).

### 3.6. Meta-Analysis of the Cho/NAA Ratio

**Figure 5:**
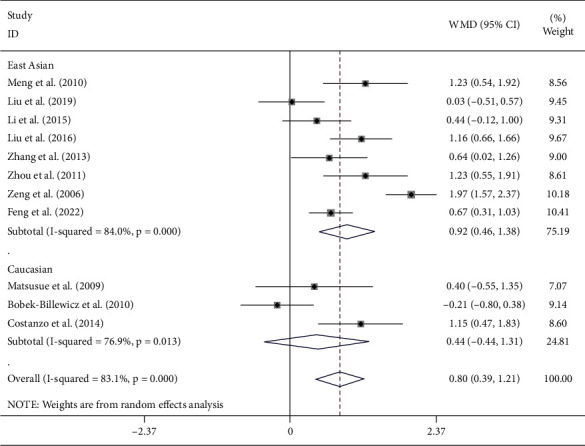
Forest plot of the Cho/NAA ratio of the radiation injury group and the recurrent glioma group detected by DWI combined with MRS.

The Cho/NAA ratio of the included studies had high heterogeneity (*I*^2^ = 83.1%, *P* < 0.001). The random-effects model was used to combine the effect sizes. The results indicated that the Cho/NAA ratio was significantly increased in the recurrent glioma group than in the radiation injury group (WMD = 0.80, 95% CI (0.39, 1.21), *P* < 0.001). Additionally, stratified analysis by ethnicity demonstrated that the abovementioned results could only be identified in East Asian groups (WMD = 0.92, 95% CI (0.46, 1.38), *P* < 0.001) but not in Caucasian populations (WMD = 0.44, 95% CI (−0.44, 1.31), *P* < 0.001) (Figure 5).

### 3.7. Meta-Analysis of the Diagnostic Efficacy of the Cho/Cr Ratio and Cho/NAA Ratio

**Figure 6:**
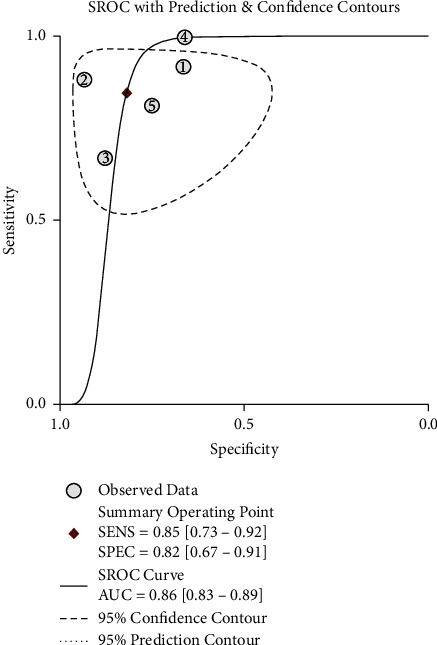
Summary ROC (SROC) curves of the diagnostic efficacy of the Cho/Cr ratio of the radiation injury group and the recurrent glioma group.

**Figure 7:**
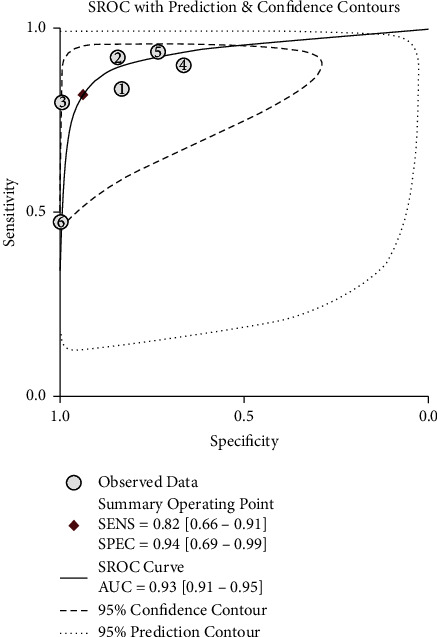
Summary ROC (SROC) curves of the diagnostic efficacy of the Cho/NAA ratio of the radiation injury group and the recurrent glioma group.

**Figure 8:**
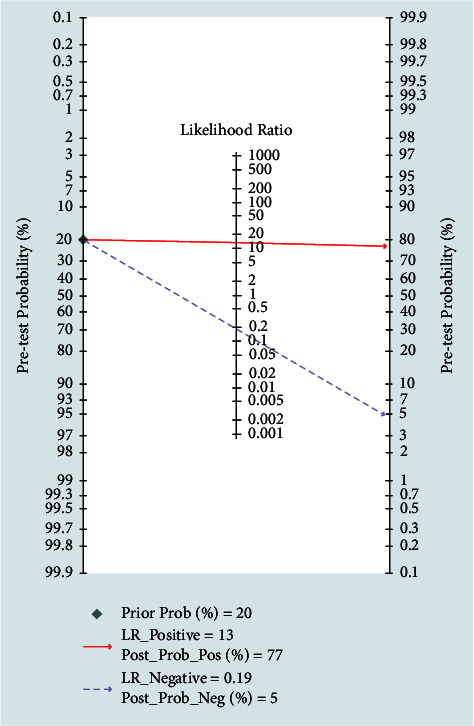
Fagan nomograms of the Cho/Cr ratio and Cho/NAA ratio of the radiation injury group and the recurrent glioma group.

The results of the meta-analysis of the diagnostic efficacy of the Cho/Cr ratio and Cho/NAA ratio are shown in Table 2 and Figures 6 and 7 (receiver operating characteristic (ROC) curves). The Cho/Cr ratio and Cho/NAA ratio had high accuracies in the differential diagnosis between recurrent glioma and radiation injury. The Fagan nomograms of the Cho/Cr ratio and Cho/NAA ratio showed that when the test result was positive, the probability of accurate detection increased from 20% (pretest probability) to 77% (post-test probability) (Figure 8). When the test result was negative, the probability of accurate detection decreased from 20% (pretest probability) to 5% (post-test probability), further indicating that the Cho/Cr ratio and Cho/NAA ratio can improve the identification efficiency of recurrent glioma and radiation injury.

### 3.8. Heterogeneity Test and Sensitivity Analysis

Significant heterogeneity between these studies was observed among the outcome measures (Figures 3–5). The results of our subgroup analysis confirmed that ethnicity was the primary sources of heterogeneity. Additionally, sensitivity analysis was conducted to evaluate the effect of an individual study on the pooled results. The pooled effects were not affected by removing any study.

### 3.9. Publication Bias

Because rADC, the Cho/Cr ratio, the Cho/NAA ratio, and other outcome measures were analysed using conventional meta-analytical methods, fewer than 10 included studies had rADC, the Cho/Cr ratio, or the Cho/NAA ratio. This meant the publication bias could not be effectively evaluated through the symmetry of the funnel plot, so it was evaluated using Egger's test. The results showed no publication bias (*P* > 0.05) in rADC (*P*=0.574), Cho/Cr ratio (*P*=0.339), and Cho/NAA ratio (*P*=0.47). The results of the Deeks funnel plots of the Cho/Cr ratio and Cho/NAA ratio did not show any publication bias in the diagnostic efficiency of the Cho/Cr ratio (*P*=0.289) or in the diagnostic efficiency of the Cho/NAA ratio (*P*=0.253).

## 4. Discussion

Glioma is characterized by diffuse growth and is the most refractory tumour with the highest recurrence rate [22, 23]. On the one hand, patients need postoperative radiotherapy to prolong their survival, but on the other hand, they must tolerate the tissue damage caused by radiotherapy. Conventional non-contrast-enhanced magnetic resonance imaging (MRI) and contrast-enhanced MRI are the most commonly used MRI techniques. However, both recurrent glioma and radiation injury show enhancement of the lesion area during enhanced MRI [4, 24] and thus are extremely difficult to differentially diagnose. In addition, the lesion area has a complex composition, with tumour cells and the radiation-damaged tissue coexisting or existing alone. The application of DWI combined with MRS in the diagnosis of recurrent glioma and radiation injury can reduce the misdiagnosis rate, thereby providing clinicians with information with which to develop treatment plans.

The results of our meta-analysis showed that ADC was lower in the recurrent glioma group than in the radiation injury group. ADC can be used to quantitatively study the diffuse movement of intracellular water molecules. Tissues of recurrent tumours have a high cell density, relatively narrow intercellular space, restricted intracellular diffusion of water molecules, and low movement ability, resulting in a decrease in ADC [25, 26]. Tissues of radiation-induced lesions have a low cell density, an enlarged intercellular space, and active diffusion of water molecules due to necrosis and liquefaction of cells and degeneration and dissolution of myelin sheaths and axons in the lesions, leading to a high ADC value [27–29]. Although ADC had high heterogeneity, sensitivity analysis and subgroup analysis did not reveal the main cause of the heterogeneity, so ADC was relatively stable. Cells in recurrent glioma proliferate vigorously and have accelerated metabolism; their cell membranes and organelles are disintegrated to release free Cho; tumour cells show infiltrating growth; and nerve cells are destroyed. The MRS manifestations of recurrent glioma include decreased NAA peaks, differently increased Cho peaks, slightly decreased Cr, and increased Cho/Cr and Cho/NAA ratios [30, 31]. Radiation-induced brain injury is a complication of radiotherapy, which is related to the radiation dose, the size of the radiation field, the radiation frequency during the treatment process, and the survival time of the patient [32]. Radiation injury can lead to cell necrosis, inflammatory repair, neuronal damage, and reduced metabolism, which manifests as the diminution or disappearance of Cho and NAA peaks. When the brain tissue is permanently damaged, the MRS parameters are all low [33, 34]. Therefore, the Cho/Cr and Cho/NAA ratios were lower in radiation injury than in recurrent glioma. The sensitivity and specificity of the Cho/Cr and Cho/NAA ratios in the diagnosis of glioma recurrence were both moderate to high.

Patients undergoing radiotherapy after glioma surgery can be diagnosed with recurrent glioma if the contrast-enhanced MRI scan reveals new enhanced lesions in the brain and the lesion area has a decreased ADC and increased Cho/Cr and Cho/NAA ratios. Otherwise, they can be diagnosed with radiation injury. Even if the lesion area contains a mixture of tumour cells and radiation-induced brain injury components, its ADC, Cho/Cr ratio, and Cho/NAA ratio will still be different from those of simple radiation injury due to the characteristics of tumour cells. Therefore, multisequence and multiparameter imaging can improve the accuracy and sensitivity of the differential diagnosis between recurrent glioma and radiation injury.

At the same time, we also note that relevant literature points out that adc, as one of the quantitative indicators of DWI, the detection of glioma is different for each tumour region, where the ADC values should be measured in the peritumoral oedema region or the gadolinium-enhanced part [35]. Of course, more clinicians are needed to test its clinical applicability. In summary, multiparameter quantitative analysis through DWI with MRS can effectively improve the accuracy of the differential diagnosis between recurrent glioma and radiation injury and provide effective imaging parameters for clinicians, thereby helping clinicians choose treatment options and improve patient prognosis.

## Figures and Tables

**Table 1:** Basic characteristics and quality evaluation of the included studies.

Author	Year	Country	Ethnicity	Study design	Comparator imaging tests	N		Outcome measures
						Glioma recurrence	Radiation injury	
Meng et al. [11]	2010	China	East Asian	Retrospective	DWI + MRS	12	6	①②③④⑤
Liu et al. [12]	2019	China	East Asian	Retrospective	DWI + MRS	15	17	①②③
Li et al. [13]	2015	China	East Asian	Retrospective	DWI + MRS	14	16	①②③
Liu and Zheng [14]	2016	China	East Asian	Retrospective	DWI + MRS	25	15	①②③④⑤
Zhang et al. [15]	2013	China	East Asian	Retrospective	DWI + MRS	12	13	①②③
Meng et al. [16]	2011	China	East Asian	Retrospective	DWI + MRS	15	7	④⑤
Matsusue et al. [17]	2010	USA	Caucasian	Retrospective	DWI + MRS	10	5	①②③④⑤
Bobek-Billewicz et al. [18]	2010	Poland	Caucasian	Retrospective	DWI + MRS	4	4	①②③
Zeng et al. [19]	2007	China	East Asian	Retrospective	DWI + MRS	32	23	①②③
Feng et al. [20]	2014	Italy	Caucasian	Retrospective	DWI + MRS	21	8	①②③
Di Costanzo et al. [21]	2022	China	East Asian	Retrospective	DWI + MRS	31	15	①②③

**Table 2:** Meta-analysis results of the diagnostic efficacies of the Cho/Cr ratio and Cho/NAA ratio.

Category	*I* ^2^ (%)		SEN (95% CI)	SPE (95% CI)	PLR (95% CI)	NLR (95% CI)	DOR (95% CI)	AUC (95% CI)
	SEN	SPE						
Cho/Cr	36.78	0.00	0.85 (0.73–0.92)	0.82 (0.67–0.91)	4.7 (2.4–9.3)	0.19 (0.10–0.35)	25 (8–75)	0.86 (0.83–0.89)
Cho/NAA	71.64	0.00	0.82 (0.66–0.91)	0.94 (0.69–0.99)	13.4 (2.4–76.1)	0.19 (0.10–0.37)	70 (13–389)	0.93 (0.91–0.95)

## Data Availability

All data and codes are available upon request to the corresponding author.
